# A-Kinase Anchor Protein 95 Is Involved in ERK1/2–Elk-1 Signal Transduction in Colon Cancer

**DOI:** 10.1155/2023/8242646

**Published:** 2023-01-14

**Authors:** Xiangyu Kong, Putian An, Junping Xu, Wenzhi Liu, Feng Lin, Yulong Yang

**Affiliations:** ^1^Center of Gallbladder Disease, Shanghai East Hospital, School of Medicine, Tongji University, Shanghai 200092, China; ^2^Affiliated Zhongshan Hospital of Dalian University, Dalian, Liaoning 116001, China

## Abstract

**Objectives:**

To assess A-kinase anchor protein 95 (AKAP95), B-Raf, extracellular regulated protein kinases 1/2 (ERK1/2), and Elk-1 expression in colon cancer tissue, and characterize AKAP95 associations with B-Raf, ERK1/2, Elk-1, and colon cancer clinicopathological indices.

**Methods:**

The immunohistochemistry streptavidin-perosidase (SP) method was used to determine protein expression levels in 64 colon cancer and 32 para-carcinoma tissue specimens.

**Results:**

(1) Positive AKAP95 expression rates in colon cancer tissue were higher when compared with para-carcinoma tissue (92.19% vs. 59.38%, *P* < 0.05). Similar findings were determined for B-Raf (76.56% vs. 25%, *P* < 0.05), ERK1/2 (90.63% vs. 31.25%, *P* < 0.05), and Elk-1 levels (92.19% vs. 40.63%, *P* < 0.05). (2) No significant associations were identified between AKAP95, B-Raf, ERK1/2, and Elk-1 protein expression and degree of differentiation, histological type, and lymph node metastasis in colon cancer samples (*P* > 0.05); however, in The Cancer Genome Atlas and Gene Expression Omnibus datasets, AKAP95 was closely related to immune infiltration, and highly expressed AKAP95 was negatively associated with overall survival and relapse free survival rates in colon cancer patients. (3) Correlations were observed between AKAP95 and ERK1/2, AKAP95 and Elk-1, B-Raf and ERK1/2, B-Raf and Elk-1, and ERK1/2 and Elk-1 (all *P* < 0.05), but no correlation was observed between AKAP95 and B-Raf (*P* > 0.05).

**Conclusions:**

AKAP95 may affect immune infiltration levels in colon cancer by participating in ERK1/2–Elk-1 signal transduction.

## 1. Background

A-kinase anchor protein 95 (AKAP95) is an anchoring protein for protein kinase A (PKA) and appears to regulate cyclic adenosine monophosphate (cAMP) accumulation by forming a distinct microdomain with PKA and phosphodiesterase (PDE4) in the nucleus [1]. We previously showed that when cAMP was activated by extracellular factors, AKAP95 was elevated and promoted cell proliferation via cyclin D/E and phospho-retinoblastoma (p-Rb) mechanisms [2–4]. Recent studies also showed that AKAP95 participated in tumorigenesis by regulating gene transcription and RNA clipping [5–7]. In addition overexpressed AKAP95 was detected in lung, ovarian, and rectal cancers [8–10]. Hence, AKAP95 is considered a cancer promoting protein; however, its role in tumorigenesis and participation in associated signaling pathways remains unclear.

The mitogen-activated protein kinase/extracellular signal-regulated (MAPK/ERK) pathway plays an important role in colorectal cancer [11]. MAPK/ERK pathway is closely related to cAMP/PKA signaling [12]. cAMP induces the sequential phosphorylation of Raf, MEK, and ERK in MAPK/ERK pathway. B-Raf is a selective target of cAMP in thyroid cells [13] and intercedes in PKA-induced ERK1/2 activation [14]. In addition, cAMP/PKA directly activates ERK [12, 13]. MAPK/ERK pathway plays an important role in transducing cAMP into the nucleus to activate Elk and other substrates [15]. However, it is unclear if AKAP95, like other AKAP family members [16–19], is involved in this signaling pathway.

Both AKAP95 and MAPK/ERK signaling pathways are critically involved in cAMP/PKA regulation [1, 12]. In this study, AKAP95, B-Raf, ERK1/2, and Elk-1 protein levels were examined in cancer and normal tissue samples, and associations between them characterized. This approach provided evidence showing that AKAP95 participated in Raf–MEK–ERK1/2 signaling to promote tumorigenesis or immune cells infiltration in colon cancer.

## 2. Materials and Methods

### 2.1. Tumor Sources

Tissue samples from 64 invasive colon cancer cases with definite pathological diagnoses were collected from the Affiliated Zhongshan Hospital of Dalian University, Dalian, China. Patient ages ranged from 51 to 82 years (average age = 70.3 ± 8.1 years) and 39 males and 25 females participated. In total, 62 patients had tubular or papillary adenocarcinoma, and two had mucinous adenocarcinoma. In 32/64 patients, para-carcinoma tissue was obtained from normal colonic tissue at least 3 cm away from cancerous tissue. Pathological examinations were also performed in para-carcinoma tissue to confirm the absence of cancer cells. Study protocols were approved by the Medical Ethics Committee of Affiliated Zhongshan Hospital of Dalian University (Ethics reference number: 2020010).

### 2.2. Reagents and Methods

Specimens were fixed in 10% neutral formaldehyde, paraffin embedded, and sliced into continuous 4 *μ*m sections. The SP-9000 immunohistochemical staining kit (Zhongshan Jinqiao Biotechnology Company, Beijing, China) was used for protein expression analyses according to manufacturer's instructions. The assay involved 3, 3′-diaminobenzidine staining and hematoxylin counterstaining. Mouse anti-human AKAP95, B-Raf, ERK1/2, and Elk-1 monoclonal antibodies were purchased from Santa Cruz (Dallas, TX, USA). Phosphate buffered saline (PBS) (pH 7.20) was used in negative control samples. Rabbit anti-AKAP8 (ab140628) was purchased from Abcam Company (Cambridge, UK); Cy3-affinipure goat anti-rabbit IgG (111-165-003) and 488-affinipure donkey anti-mouse IgG (715-545-150) were purchased from Jackson ImmunoResearch Laboratories Inc. (West Grove, PA, USA); Protein A/G Plus-Agarose (sc-2003) was from Santa Cruz; Cell lysis buffer for Western blot and Immunoprecipitation (IP) (P0013) were purchased from Beyotime Institute of Biotechnology (Haimen, China).

### 2.3. Co-Immunoprecipitation (Co-IP)

When HCT116 cells cultured to 80% confluence, cells were collected and lysed with the WB/IP lysate buffer. 500 *μ*g proteins were incubated with the antibody for 1 hour on a shaker, then incubated with Protein A/G Plus-Agarose overnight. The precipitant was centrifuged and washed three times in PBS in 4°C, then resuspended by using a sample buffer and identified by western blot assay.

### 2.4. Western Blot Assay

Proteins were Sodium Dodecyl Sulfate PolyAcrylamide Gel Electrophoresis (SDS-PAGE) separated, electro-transferred to a membrane, and then incubated with primary antibody at 4°C overnight, incubated with secondary antibody at room temperature for 1 hour, Enhanced Chemiluminescence (ECL)-developed, exposed, and imaged using the Tanon-4600SF Imaging System (Shanghai, China).

### 2.5. Immunofluorescence

HCT116 cells grown on slides were treated with 0.5% Triton-X 100 at room temperature for 30 minutes, blocked in 3% bovine serum albumin (BSA), incubated with primary antibody at 4°C overnight, and labeled by CY-3 and 488 fluorescent antibody (at a dilution of 1 : 300) at 37°C in a dark room. Nuclei were counter-stained by 4′,6-Diamidino-2′-phenylindole (DAPI), and smears were observed under a fluorescence microscope (BX53, Olympus, Tokyo, Japan).

### 2.6. Criteria Indicating Positive Protein Expression

A brown-yellow stain indicated positive protein expression, whereas its absence indicated no protein expression. Ten different fields/sections were randomly evaluated, with 200 tumor cells counted/field. Positive total cell ratios were used as metrics to assess positive protein expression: “−” indicated that <10% of cancer cells were yellow or brown; “±” indicated that ≥10% and <25% of cancer cells were yellow or brown; “+” indicated that ≥25% and <50% of cancer cells were yellow or brown; “++” indicated that ≥50% and <75% of cancer cells were yellow or brown; and “+++” indicated that >75% of cancer cells were yellow or brown.

For data analyses, “−” and “±” indicated negative expression. In addition “+” indicated low expression, “++” indicated moderate expression, and “+++” indicated high expression levels; thus, all indicated positive expression.

### 2.7. Bioinformatics and Functional Enrichment Analysis

Genomic data of colon cancer patient were collected from The Cancer Genome Atlas (TCGA) and Gene Expression Omnibus (GEO) databases. We analyzed the relationship between AKAP95 (AKAP8) expression and overall survival (OS) and relapse free survival (RFS) in colon cancer patients among these datasets. The data of relationship between AKAP95 (AKAP8) and tumor infiltrates immune cells were analyzed with methods including CIBERSORT, CIBERSORT-ABS, EPIC, ESTIMATE, MCPCOUNTER, QUANTISEQ, TIMER, and XCELL [20, 21].

### 2.8. Statistical Analysis

The SPSS20.0 software (SPSS Inc., Chicago, IL, USA) was used for statistical analysis.

Expression ratio comparisons were performed using Chi-square tests, and protein expression correlation analyses were performed using Spearman's rank correlations. A *P* < 0.05 value indicated statistical significance [22, 23].

## 3. Results

### 3.1. AKAP95, B-Raf, ERK1/2, and ELK-1 Expression Levels in Colon Cancer Tissue

**Figure 1:**
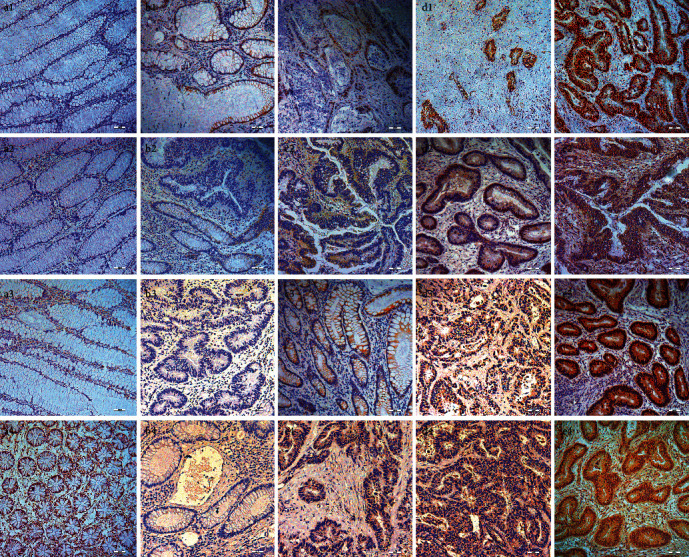
Representative images showing AKAP95, B-Raf, ERK1/2, and ELK-1 expressions in colon cancer and para-carcinoma tissue (magnification = 10×). (a1) No AKAP95 expression in para-carcinoma tissue. (b1) AKAP95 showing some expression (− or ±) in colon cancer tissue. (c1) Lowly expressed AKAP95 (+) in colon cancer tissue. (d1) Moderately expressed AKAP95 (++) in colon cancer tissue. (e1) Highly expressed AKAP95 (+++) in colon cancer tissue. (d1) and (e1) AKAP95 expression not only in the nuclei, but also in the cytoplasm. (a2) No B-Raf expression in para-carcinoma tissue. (b2) B-Raf showing some expression (− or ±) in colon cancer tissue. (c2) Lowly expressed B-Raf (+) in colon cancer tissue. (d2) Moderately expressed B-Raf (++) in colon cancer tissue. (e2) Highly expressed B-Raf (+++) in colon cancer tissue. (c2)–(e2) B-Raf expression in the cytoplasm. (a3) No ERK1/2 expression in para-carcinoma tissue. (b3) ERK1/2 showing some expression (− or ±) in colon cancer tissue. (c3) Lowly expressed ERK1/2 (+) in colon cancer tissue. (d3) Moderately expressed ERK1/2 (++) in colon cancer tissue. (e3) Highly expressed ERK1/2 (+++) in colon cancer tissue. (c3) ERK1/2 mainly expression in the cytoplasm. (d3) and (e3) ERK1/2 expression in the cytoplasm and nucleus. (a4) No ELK-1 expression in para-carcinoma tissue. (b4) ELK-1 showing some expression (− or ±) in colon cancer tissue. (c4) Lowly expressed ELK-1 (+) in colon cancer tissue. (d4) Moderately expressed ELK-1 (++) in colon cancer tissue. (e4) Highly expressed ELK-1 (+++) in colon cancer tissue. (c4) and (d4) ELK mainly expression in the cytoplasm. (e4) ELK-1 expression in the cytoplasm and nucleus.

AKAP95, ERK1/2, ELK-1, and B-Raf expression levels in 64 colon cancer and 32 para-carcinoma samples were assessed (Table 1). AKAP95 positive rates were 92.19% in colon cancer (Table 1; Figures 1(b1), 1(c1), 1(d1), and 1(e1)) and 59.38% in para-carcinoma specimens (Table 1, Figure 1(a1)). B-Raf positive rates were 76.56% in colon cancer (Table 1; Figures 1(b2), 1(c2), 1(d2), and 1(e2)) and 25% in para-carcinoma specimens (Table 1; Figure 1(a2)). ERK1/2 positive rates were 90.63% in colon cancer (Table 1; Figures 1(b3), 1(c3), 1(d3), and 1(e3)) and 31.25% in para-carcinoma specimens (Table 1; Figure 1(a3)). Elk-1 positive rates were 92.19% in colon cancer (Table 1; Figures 1(b4), 1(c4), 1(d4), and 1(e4)) and 40.63% in para-carcinoma specimens (Table 1; Figure 1(a4)). Differences were all statistically significant (Table 1; all *P* < 0.001). We also observed that AKAP95 (Figures 1(d1) and 1(e1)) and Elk-1 (Figure 1(e4)) were mainly expressed in nuclei in colon carcinoma tissue, whereas B-Raf (Figures 1(c2), 1(d2), and 1(e2)) and ERK1/2 (Figures 1(c3), 1(d3), and 1(e3)) subcellular localization were predominantly cytoplasmic.

### 3.2. Relationships between Clinical Pathological Parameters or Immune Cell Infiltration and AKAP95, ERK1/2, Elk-1, and B-Raf Expression

We observed no AKAP95 associations with tumor-node-metastasis stages, degree of differentiation, vascular invasion, lymph node metastasis, and distant metastasis (Table 2; *P* > 0.05). ERK1/2, Elk-1, and B-Raf also showed similar results (Supplemental Tables S1, S2, and S3). When we analyzed TCGA and GEO datasets, elevated AKAP95 expression was negatively associated with OS and RFS in colon cancer patients (Supplemental Figure S1(a) and (b)).

Since tumor infiltration by immune cells is a vital parameter for patient survival, correlations between AKAP95 and immune cells were also analyzed in TCGA and GEO datasets (Supplemental Figure S2(a)). AKAP95 showed positive relationships with CD4 naïve T cells, M0 macrophages, and resting dendritic cells (Supplemental Figures S2(b), (c), (d) and (e)). AKAP95 showed negative relationships with Immune Score and ESTIMATE Score (Supplemental Figures S2(f) and (g)).

### 3.3. AKAP95, ERK1/2, Elk-1, and B-Raf Associations in Colon Cancer Tissue

We also analyzed associations between AKAP95 and B-Raf, ERK1/2, and Elk-1 in colon cancer tissue. We identified significant correlations between AKAP95 and ERK1/2 (*P* < 0.05; Table 3) and AKAP95 and ELK-1 (*P* < 0.05; Table 4). No significant associations were identified between AKAP95 and B-Raf (Table 5; *P* > 0.05). Additionally, significant correlations were identified between ERK1/2 and B-Raf (Table 6; *P* < 0.05), ERK1/2 and Elk-1 (Table 7; *P* < 0.05), and Elk-1 and B-Raf (Table 8; *P* < 0.05).

In order to study the relationship between AKAP95 and ERK1/2 protein, Co-IP and immunofluorescence experiments were performed. The results showed that AKAP95 bound with ERK1/2 (Supplemental Figures S3(a) and (b)), and they were co-localized in HCT116 cells (Supplemental Figure S3(c)).

## 4. Discussion

AKAP95 promotes cancer cell growth and is highly expressed in lung, rectal, esophageal, ovarian, and breast cancers [4–7]. AKAP95 mechanisms suggest participation in cell cycle regulation by affecting cyclin D/E expression [4, 7]. Moreover, AKAP95 suppresses oncogene-induced senescence by regulating transcription and RNA splicing [10], and which play an important role in tumorigenesis [10]. In our study, the positive AKAP95 protein expression rate was 92.19% (59/64) in colon cancer and 59.38% (19/32) in para-carcinoma specimens, which suggested a functional role for AKAP95 in tumorigenesis. Otherwise, AKAP95 expressed not only in the nuclei but in cytoplasm of colon cancer was also found in present study (Figures 1(d1) and 1(e1)), which was as the same as our previous results [12]. This observation suggested that AKAP95 not only had important roles in the nucleus but was also important in the cytoplasm during tumorigenesis.

AKAP95 participates in cAMP signal transduction by anchoring the RII subunit of PKA [24]. cAMP, which is produced after extracellular receptor stimulation, may regulate cyclin D by ERK1/2 signaling [25, 26]. When cAMP was increased using forskolin, AKPA95 levels were increased and the protein bound to cyclin D/E to promote cell proliferation [3, 4]. In this study, AKAP95 expression was positively correlated with ERK1/2 levels in colon cancer specimens (Table 3). In addition, the results showed that AKAP95 and ERK1/2 bound together in HCT116 cells (Supplemental Figure S3). Therefore, we hypothesize that AKAP95, like its family members, participates in ERK signal transduction. For example, AKAP-Lbc binds to the ERK scaffold protein KSR-1 and forms a growth factor and cAMP reactive signal network to transmit signals from Raf to ERK1/2 via MEK [16, 17]. AKAP79 transmits signals generated by inhibitory GTP-binding protein (Gi) coupled to the beta2-adrenoceptor (*β*2-AR) receptor and to ERK [18]. mAKAP-*β* transmits extracellular signals from Angiotensin II (AngII) induced cardiomyocyte hypertrophy through phosphor-extracellular regulated protein kinases p-ERK2 [19]. However, in this current study, AKAP95 was not significantly correlated with B-Raf, but positively correlated with ERK1/2 and Elk-1. Therefore, AKAP95 may be involved in ERK1/2 signal transduction to Elk-1 by binding to ERK1/2 proteins. However, the results need to design detailed experiments to verify in future.

The transcription factor, Elk-1, is a nuclear substrate of ERK. When the ERK/Elk-1/Snail pathway was activated, it promoted epithelial–mesenchymal transition in colon cells and lung tissues [15]. The ERK pathway has also important roles in tumor immune invasion; however, AKAP95, ERK1/2, and Elk-1 expressions were not related to colon cancer invasion and metastasis but were possibly related to our low sample numbers. Fortunately, by using TCGA and GEO datasets, AKAP95 was closely related to tumor immune invasion, and high AKAP95 expression affected OS and tumor free survival rates in the GSE106584 dataset (Supplemental Figures S1 and S2). However, to comprehensively verify these observations, further studies are required.

In conclusion, AKAP95 was closely correlated with Raf–MEK–ERK signaling and was putatively involved in signal transduction from ERK to Elk so as to affect immune cell infiltration, resulting in affecting OS and RFS in colon cancer patients.

## Figures and Tables

**Table 1:** AKAP95, ERK1/2, ELK-1, and B-Raf protein levels in colon cancer and normal tissue.

Protein	Status	Colon cancer	Normal tissue	*χ* ^2^	*P*-value
AKAP95	Positive	59	19	15.07	<0.001
	Negative	5	13		
B-Raf	Positive	49	8	23.51	<0.001
	Negative	15	24		
ERK1/2	Positive	58	10	36.40	<0.001
	Negative	6	22		
ELK-1	Positive	59	13	30.25	<0.001
	Negative	5	19		

*χ* ^2^: chi-square tests.

**Table 2:** AKAP95 protein expression associations with clinical–pathological parameters.

Item	AKAP95 status		*χ* ^2^	*P*-value
	Positive	Negative		
TNM stage				
T1–T2	31	1	1.95	0.16
T3–T4	28	4		
Differentiation				
High	1	0	0.76	0.68
Moderate	51	5		
Low	7	0		
T stage				
T1–T2	3	0	0.27	0.61
T3–T4	56	5		
Lymph node
Yes	31	2	0.29	0.59
No	28	3		
Metastasis
Yes	4	0	0.93	0.54
No	55	5		
Vascular invasion
Yes	17	0	1.96	0.16
No	42	5		

*χ* ^2^: chi-square tests. TNM stage: tumor node metastasis staging classification; differentiation: differentiation degree of colon cancer cells; T stage: local invasion of colon cancer cells; lymph node: colon cancer cells metastasis in regional lymph node; metastasis: colon cancer cells distant metastasis; vascular invasion: colon cancer cells invasion vascular, lymphatic vessel, or neural invasion.

**Table 3:** The relationship between AKAP95 and ERK1/2 protein expression in colon cancer tissue.

ERK1/2	AKAP95					*r*_s_	*P*-value
	−	±	+	++	+++		
−	1	1	1	0	1	0.265	0.034
±	0	0	1	0	1		
+	1	2	1	1	6		
++	0	0	1	1	12		
+++	0	0	0	10	23		

*r*_s_: Spearman's rank correlation's coefficient.

**Table 4:** The relationship between AKAP95 and ELK-1 protein expression in colon cancer tissue.

ELK-1	AKAP95					*r*_s_	*P*-value
	−	±	+	++	+++		
−	0	1	1	0	2	0.252	0.045
±	1	0	0	0	0		
+	0	2	1	4	10		
++	1	0	1	2	10		
+++	0	0	1	6	21		

*r*_s_: Spearman's rank correlation's coefficient.

**Table 5:** The relationship between AKAP95 and B-Raf protein expression in colon cancer tissue.

B-Raf	AKAP95					*r*_s_	*P*-value
	−	±	+	++	+++		
−	0	2	1	0	5	0.182	0.149
±	0	1	1	1	4		
+	1	0	1	2	9		
++	1	0	1	5	10		
+++	0	0	0	4	15		

*r*_s_: Spearman's rank correlation's coefficient.

**Table 6:** The relationship between B-Raf and ERK1/2 protein expression in colon cancer tissue.

B-Raf	ERK1/2					*r*_s_	*P*-value
	−	±	+	++	+++		
−	1	1	3	3	0	0.603	<0.001
±	2	0	3	1	1		
+	0	0	4	2	7		
++	1	1	1	6	8		
+++	0	0	0	2	17		

*r*_s_: Spearman's rank correlation's coefficient.

**Table 7:** The relationship between ERK1/2 and ELK-1 protein expression in colon cancer tissue.

ELK-1	ERK1/2					*r*_s_	*P*-value
	−	±	+	++	+++		
−	1	0	2	1	0	0.421	0.001
±	1	0	0	0	0		
+	2	1	3	4	7		
++	0	0	3	5	6		
+++	0	1	3	4	20		

*r*_s_: Spearman's rank correlation's coefficient.

**Table 8:** The relationship between B-Raf and ELK-1 protein expression in colon cancer tissue.

B-Raf	ELK-1					*r*_s_	*P*-value
	−	±	+	++	+++		
−	2	0	2	2	2	0.257	0.041
±	0	0	4	1	2		
+	2	0	2	4	5		
++	0	1	5	2	9		
+++	0	0	4	5	10		

*r*_s_: Spearman's rank correlation's coefficient.

## Data Availability

Datasets analyzed in this work can be obtained from TCGA (https://portal.gdc.cancer.gov/) and GEO (https://www.ncbi.nlm.nih.gov/geo/).

## Supplementary Materials

Supplementary Materials Tables S1, S2, and S3 showed ERK1/2, ELK-1, and B-Raf protein expression associations with TNM stages, degree of differentiation, vascular invasion, lymph node metastasis, and distant metastasis, respectively. Figure S1 showed AKAP95 expression was negatively associated with OS and RFS in colon cancer patients in TCGA and GEO datasets. Figure S2 showed the relationships between AKAP95 expression and immune cells infiltrating the tumor in TCGA and GEO datasets. Figure S3 showed the relationship between AKAP95 and ERK1/2 protein by co-immunoprecipitation and immunofluorescence in HCT116 cells.
