# Supplementary material for: A-Kinase Anchor Protein 95 Is Involved in ERK1/2–Elk-1 Signal Transduction in Colon Cancer
Source: Anal Cell Pathol (Amst). 2023 Jan 14;2023:8242646. doi: 10.1155/2023/8242646 (PMC9867590; doi:10.1155/2023/8242646)

**Table S1.** ERK1/2 protein expression associations with clinical-pathological parameters.

Note: χ2 = Chi-square tests. TNM stage = tumor node metastasis staging classification; Differentiation = Differentiation degree of colon cancer cells; T stage= Local invasion of colon cancer cells; Lymph node= colon cancer cells metastasis in regional lymph node; Metastasis= Colon cancer cells distant metastasis; Vascular invasion= colon cancer cells invasion vascular, lymphatic vessel or neural invasion.

**Table S2.** ELK-1 protein expression associations with clinical-pathological parameters.

Note: χ2 = Chi-square tests. TNM stage = tumor node metastasis staging classification; Differentiation = Differentiation degree of colon cancer cells; T stage= Local invasion of colon cancer cells; Lymph node= colon cancer cells metastasis in regional lymph node; Metastasis= Colon cancer cells distant metastasis; Vascular invasion= colon cancer cells invasion vascular, lymphatic vessel or neural invasion.

**Table S3.** B-Raf protein expression associations with clinical-pathological parameters.

Note: χ2 = Chi-square tests. TNM stage = tumor node metastasis staging classification; Differentiation = Differentiation degree of colon cancer cells; T stage= Local invasion of colon cancer cells; Lymph node= colon cancer cells metastasis in regional lymph node; Metastasis= Colon cancer cells distant metastasis; Vascular invasion= colon cancer cells invasion vascular, lymphatic vessel or neural invasion.

**Figure S1:** Association between AKAP95 (AKAP8) with prognosis. (A) Relationship between AKAP95 (AKAP8) expression and OS in GSE106584. (B) Relationship between AKAP95 (AKAP8) expression and RSF in GSE106584.

**Figure S2:** Association between AKAP95 (AKAP8) with immune cell infiltration. A) Correlation of AKAP95 (AKAP8) between different immune cells infiltration in GSE21510, GSE106584, GSE71187, GSE10928, TCGA-CRC, GSE87211, GSE25071, GSE26682, GSE28722, GSE104645. B) Correlation between AKAP95 (AKAP8) and CD4 naive T cells. C, D) Correlation between AKAP95 (AKAP8) and M0 macrophages. E) Correlation between AKAP95 (AKAP8) and resting dendritic cells. F, G) Correlation between AKAP95 (AKAP8) with Immune Score, and ESTIMATE Score.

**Figure S3:** Determination of the interaction between AKAP95 and ERK1/2 in HCT116 cells (n = 3). A) AKAP95 antibodies was used for Co-Immunoprecipitation, then ERK1/2 and AKAP95 were detected by Western blot. AKAP95, ERK1/2 was shown in the inputs and in the AKAP95 immunoprecipitated product. B) ERK1/2 antibodies was used for Co-Immunoprecipitation, and AKAP95, ERK1/2 were detected by Western blot. AKAP95, ERK1/2 was shown in the inputs and in the ERK1/2 immunoprecipitated product. C) Co-localization of AKAP95, ERK1/2 was detected by immunofluorescence in HCT116 cells. Blue fluorescence represents DAPI-labeled nuclei; Red fluorescence represents Cy3-labeled AKAP95; Green fluorescence represents 488-labeled ERK1/2; Yellow fluorescence represents Co-localization of AKAP95, ERK1/2; Merged image was AKAP95, ERK1/2 and nuclei.

| Item | B-Raf | | | χ^2^ | P | |
| --- | --- | --- | --- | --- | --- | --- |
|  | Positive | | Negtive |  |  |  |
| TNM satge |  | |  |  | | |
| T 1-2 | 24 | | 8 | 0.08 | 0.76 | |
| T 3-4 | 25 | | 7 |  |  |  |
| Differentiat |  | | |  | | |
| High | 1 | | 0 | 0.71 | 0.70 | |
| Moderate | 42 | | 14 |  |  |  |
| Low | 6 | | 1 |  |  |  |
| T satge |  |  | |  | | |
| T 1-2 | 3 | | 0 | 0.96 | 0.33 | |
| T 3-4 | 46 | | 15 |  |  |  |
| Lymph node |  | | |  | | |
| Yes | 25 | | 8 | 0.02 | 0.87 | |
| No | 24 | | 7 |  |  |  |
| Metastasis |  | | |  | | |
| Yes | 4 | | 0 | 1.22 | 0.26 | |
| No | 45 | | 15 |  |  |  |
| vascular invasion |  |  | |  | |  |
| Yes | 12 | | 5 | 0.47 | 0.50 | |
| No | 37 | | 10 |  |  |  |

**Table S1.** ERK1/2 protein expression associations with clinical-pathological parameters

| Item | ERK1/2 | | | χ^2^ | P | |
| --- | --- | --- | --- | --- | --- | --- |
|  | Positive | | Negtive |  |  |  |
| TNM satge |  | |  |  | | |
| T 1-2 | 28 | | 4 | 0.73 | 0.39 | |
| T 3-4 | 30 | | 2 |  |  |  |
| Differentiat |  | | |  | | |
| High | 1 | | 0 | 0.32 | 0.85 | |
| Moderate | 52 | | 5 |  |  |  |
| Low | 6 | | 1 |  |  |  |
| T satge |  |  | |  | | |
| T 1-2 | 3 | | 0 | 0.32 | 0.57 | |
| T 3-4 | 55 | | 6 |  |  |  |
| Lymph node |  | | |  | | |
| Yes | 30 | | 3 | 0.01 | 0.93 | |
| No | 28 | | 3 |  |  |  |
| Metastasis |  | | |  | | |
| Yes | 4 | | 0 | 0.43 | 0.51 | |
| No | 54 | | 6 |  |  |  |
| vascular invasion |  |  | |  | |  |
| Yes | 16 | | 1 | 0.33 | 0.56 | |
| No | 42 | | 5 |  |  |  |

**Table S2.**  ELK-1 protein expression associations with clinical-pathological parameters

| Item | ELK-1 | | | χ^2^ | P | |
| --- | --- | --- | --- | --- | --- | --- |
|  | Positive | | Negtive |  |  |  |
| TNM satge |  | |  |  | | |
| T 1-2 | 30 | | 2 | 0.21 | 0.64 | |
| T 3-4 | 29 | | 3 |  |  |  |
| Differentiat |  | | |  | | |
| High | 1 | | 0 | 0.78 | 0.68 | |
| Moderate | 51 | | 5 |  |  |  |
| Low | 7 | | 1 |  |  |  |
| T satge |  |  | |  | | |
| T 1-2 | 3 | | 0 | 0.32 | 0.57 | |
| T 3-4 | 56 | | 5 |  |  |  |
| Lymph node |  | | |  | | |
| Yes | 31 | | 2 | 0.29 | 0.59 | |
| No | 28 | | 3 |  |  |  |
| Metastasis |  | | |  | | |
| Yes | 4 | | 0 | 0.93 | 0.54 | |
| No | 55 | | 5 |  |  |  |
| vascular invasion |  |  | |  | |  |
| Yes | 17 | | 1 | 1.92 | 0.16 | |
| No | 42 | | 5 |  |  |  |

**Table S3.**  B-Raf protein expression associations with clinical-pathological parameters

**Figure S1**


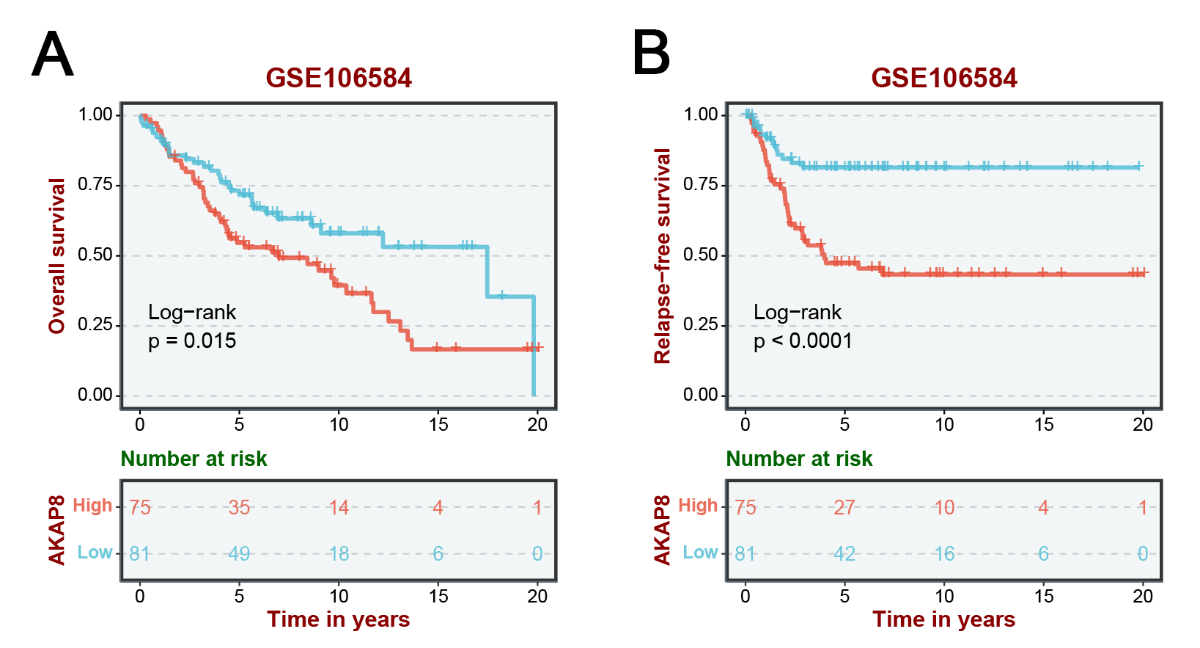


**Figure S2**


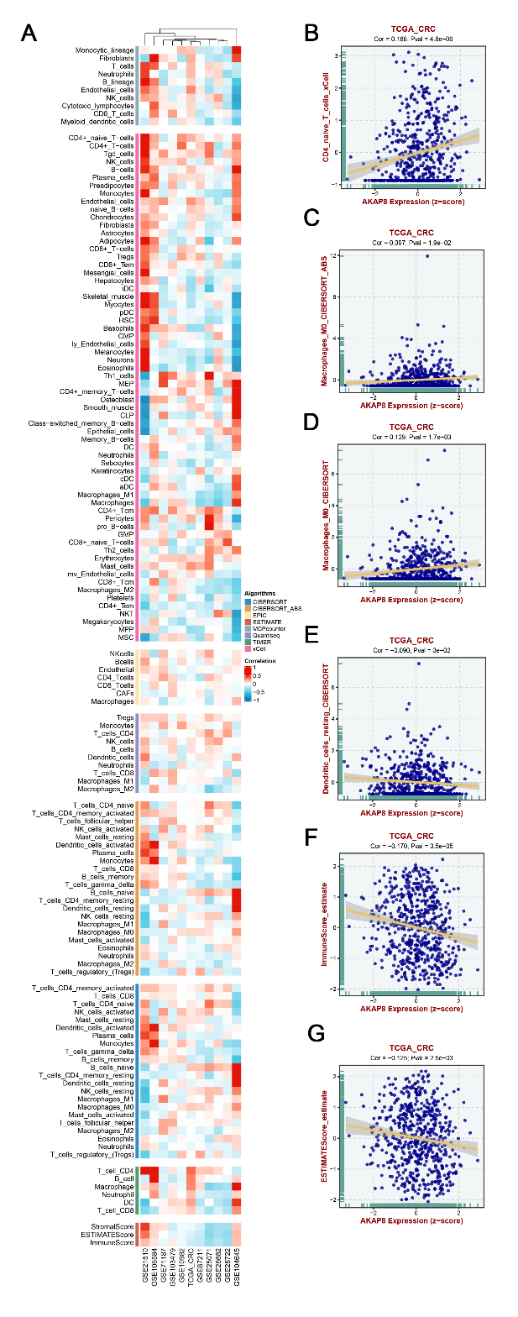


**Figure S3:**


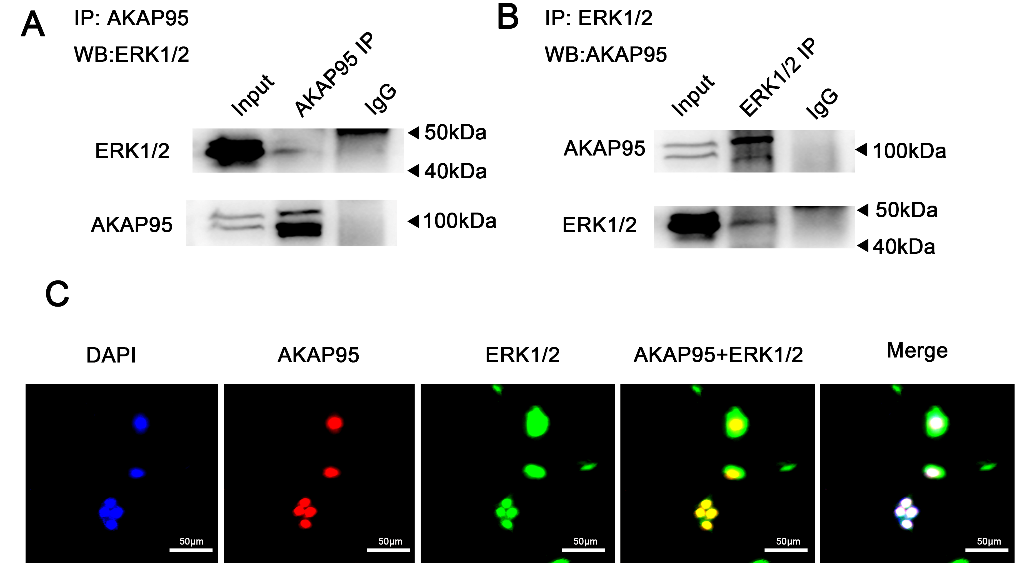

Supplement: Supplementary Materials — Tables S1, S2, and S3 showed ERK1/2, ELK-1, and B-Raf protein expression associations with TNM stages, degree of differentiation, vascular invasion, lymph node metastasis, and distant metastasis, respectively. Figure S1 showed AKAP95 expression was negatively associated with OS and RFS in colon cancer patients in TCGA and GEO datasets. Figure S2 showed the relationships between AKAP95 expression and immune cells infiltrating the tumor in TCGA and GEO datasets. Figure S3 showed the relationship between AKAP95 and ERK1/2 protein by co-immunoprecipitation and immunofluorescence in HCT116 cells. [file 8242646.f1.docx]
